# Factors Associated with Metabolically Unhealthy Obesity and Its Relation to Food Insecurity in Korean Adults with Obesity

**DOI:** 10.3390/nu16223833

**Published:** 2024-11-08

**Authors:** Jimin Lee, Wonsock Kim, Jae-Min Park, Youn Huh, Jung Hwan Kim, Young Sik Kim, Seo Young Kang

**Affiliations:** 1 Eulji University School of Medicine, Daejeon 34824, Republic of Korea; 2 Department of Family Medicine, Uijeongbu Eulji Medical Center, Uijeongbu 11759, Republic of Korea; 3 Department of Family Medicine, Gangnam Eulji Medical Center, Seoul 06047, Republic of Korea; 4 Department of Family Medicine, Asan Medical Center, University of Ulsan College of Medicine, Seoul 05505, Republic of Korea

**Keywords:** food insecurity, obesity, metabolic disease

## Abstract

Objectives: The association between food insecurity and metabolically unhealthy obesity (MUO) in the population with obesity is unclear. We evaluated factors associated with MUO and the relationship between food insecurity and MUO in individuals with obesity. Methods: We analyzed data from 5191 adults with obesity (body mass index ≥ 25 kg/m^2^) from the 8th Korea National Health and Nutrition Examination Survey 2019–2021. MUO was defined when participants with obesity had any of the following: (1) triglycerides ≥ 150 mg/dL, (2) High-density lipoprotein-cholesterol < 40 mg/dL (men), <50 mg/dL (women), (3) systolic blood pressure ≥ 135 mmHg, diastolic blood pressure ≥85 mmHg or on treatment for hypertension, (4) fasting glucose ≥ 100 mg/dL, or on treatment for diabetes. The odds ratios (ORs) and 95% confidence intervals (CIs) for MUO according to food security status, sociodemographic characteristics, and lifestyle factors were calculated using multivariate logistic regression analysis. Results: The prevalence of MUO and metabolically healthy obesity (MHO) among the participants was 85.4% and 14.6%, respectively. In the multivariate model, the OR (95% CIs) for MUO in the food insecurity group was 1.87 (1.03–3.43). The odds for MUO were higher among participants with older age, higher BMI, <12 years of education, lower fat intake, non-manual work, and moderated and low physical activity than among their counterparts. Conclusions: Food insecurity, older age, higher BMI, lower educational level, lower fat intake, non-manual workers, and lower physical activity were associated with MUO. Therefore, targeted interventions and policies are needed for vulnerable groups.

## 1. Introduction

Obesity is a critical global health issue and a significant risk factor for coronary heart disease, hypertension, stroke, type 2 diabetes, dyslipidemia, cancer, and other non-communicable diseases (NCDs) [1,2]. It reduces the quality of life and increases medical and socioeconomic costs, burdening the healthcare system [2]. The worldwide prevalence of obesity has more than tripled between 1975 and 2022, likely due to urbanization, sedentary lifestyle, and increased consumption of high-calorie processed foods [3]. Reflecting global patterns, the prevalence of obesity in South Korea has also risen from 30.2% to 38.4% from 2012 to 2021 [4].

The concept of metabolically healthy obesity (MHO) emerged from clinical observations and research findings in the 1990s and early 2000s, which led to the recognition that not all individuals with obesity share the same metabolic risks. There is no universally accepted definition of MHO, leading to variability in research findings [5,6]. However, common criteria used to define MHO include the components of metabolic syndrome, such as alteration in blood pressure, fasting blood glucose levels, triglyceride levels, and high-density lipoprotein (HDL) cholesterol levels. MHO was assumed to be a stable phenotype, but more recent research suggests that it is a transient state, and some individuals may progress to metabolically unhealthy obesity (MUO) over time [7,8]. In addition to poor cardiometabolic parameters, certain socioeconomic and lifestyle factors, including older age, being a male, lower economic status, smoking, and heavy drinking, were associated with MUO or a transition to MUO in previous studies [9,10]. Furthermore, several genetic and biological factors, including increased expression of genes involved in BCAA catabolism and lipogenesis and decreased expression of genes involved in inflammation, were associated with the development of MUO [11].

Food insecurity is defined as “a household-level economic and social condition characterized by limited or uncertain access to adequate food” [12]. Food security is a concept that includes all of the availability, access, utilization, and stability of food [13]. A paradoxical association between obesity and food insecurity has been studied over the past decades. Meta-analyses on the relationship between food insecurity and adult weight abnormalities suggest that severe food insecurity is more strongly associated with a higher risk of being underweight compared to moderate food insecurity [14]. Additionally, a lower level of national economic development is more strongly linked to severe food insecurity, whereas higher levels of national economic development are associated with moderate food insecurity, which increases the risk of obesity [14]. For instance, Africa, where food insecurity is most severe, has the highest prevalence of underweight individuals among all continents [15,16]. Conversely, in developed regions such as North America and Europe, food insecurity often leads to nutrient-deficient diets rich in inexpensive, energy-dense foods high in fats and sugars, contributing to obesity [14,15,16].

Although there are many studies focusing on MUO, few studies have been conducted to investigate the associated factors for MUO in the Korean population with obesity. Furthermore, studies focusing on the association between metabolic health and food insecurity in populations with obesity are lacking. Therefore, in this study, we examined the factors associated with MUO in Korean adults with obesity with an additional analysis of food insecurity.

## 2. Materials and Methods

### 2.1. Study Population

We analyzed data from the 8th Korea National Health and Nutrition Examination Survey (KNHANES) from 2019 to 2021. The KNHANES is a comprehensive survey conducted by the Korea Centers for Disease Control and Prevention Agency (KDCA) to assess the health and nutritional status of the South Korean population. This is a nationally representative survey that has been conducted periodically since 1998. The KNHANES uses a complex multistage sampling design to ensure that the survey results are representative of the entire South Korean population. The KNHANES was approved by the institutional review board of the KDCA, and all participants provided informed consent. The institutional review board of Eulji University provided exempt approval for this study (UEMC 2024-08-002) because the KNHANES data do not contain identifying information about participants. Of the 22,559 participants in the 8th KNHANES, we excluded those under 19 years of age, with BMI <25 kg/m^2^, and without laboratory values. We also excluded those who had not responded to lifestyle factors or levels of food security. A total of 5191 participants were included in the analysis.

### 2.2. Sociodemographic, Lifestyle, and Anthropometric Variables

Sociodemographic variables such as age, sex, household income, education, and occupation were collected. Age was sectioned into three categories: 19–39 years, 40–59 years, and ≥60 years. Household income was categorized into quartiles, which were low (1st quartile), middle (2nd and 3rd quartiles), and high (4th quartile). Educational levels were divided into <12 years and ≥12 years. Initially, occupations were categorized into ten groups based on the Korean Standard Classification of Occupations: managers, professionals, clerks, service workers, salespersons, skilled workers in agriculture/forestry/fishery, craftsmen/tradespeople, equipment/machine operators, assembly workers, elementary workers, and armed forces personnel [17]. Non-manual workers included managers, professionals, and clerks, whereas the remaining categories were considered manual workers. Subsequently, participants were categorized into non-manual workers, manual workers, and non-occupation groups.

Alcohol consumption status was classified based on the criteria of the National Institute on Alcohol Abuse and Alcoholism [18]. For men under 65 years of age, heavy drinking was defined as consuming more than fourteen standard glasses per week, and moderate drinking was defined as consuming between one and fourteen standard glasses per week. For men aged ≥65 years and women under 65 years, heavy drinking was defined as consuming over seven standard glasses per week, and moderate drinking was defined as consuming between one and seven standard glasses per week. For women aged ≥65 years, heavy drinking was defined as consuming more than three standard glasses per week, and moderate drinking was defined as consuming one to three standard glasses per week. Subsequently, alcohol consumption was grouped into non-drinkers, moderate drinkers, or heavy drinkers. Smokers were categorized as never-smokers, former smokers, or current smokers.

Physical activity was calculated using the Global Physical Activity Questionnaire and categorized as high, moderate, and low [19]. Regarding eating behavior, eating out was categorized as <1 time/day and ≥1 time/day. Excessive calorie intake was calculated based on adequate calorie intake considering age and sex [20]. High-fat intake was defined when fat intake was ≥30% of the energy intake.

BMI was calculated by dividing a person’s weight (kg) by their height squared (m^2^). Height was recorded with precision to the nearest 0.1 cm, whereas weight was measured with accuracy to the nearest 0.1 kg. In this study, obesity was defined as BMI ≥ 25 kg/m^2^ according to the guidelines of the World Health Organization Asia-Pacific Region and the Korean Society for the Study of Obesity [21,22].

Systolic blood pressure (SBP) and diastolic blood pressure (DBP) were both assessed three times, spaced 5 min apart. The last two SBP and DBP measurements of SBP and DBP were averaged, and the average values were used in this study. Participants who had fasted for at least 8 h provided blood samples, which were then analyzed by a certified laboratory to determine levels of fasting glucose, HbA1c, total cholesterol, low-density lipoprotein-cholesterol (LDL), HDL, triglycerides, aspartate transaminase (AST), and alanine transaminase (ALT).

### 2.3. Measurement of Food Insecurity

Household food security status was assessed according to an 18-item questionnaire tailored for Koreans, adapted from the United States Household Food Security Survey Module (US HFSSM) [23]. This questionnaire gauged the dietary experiences stemming from financial constraints over the past year. Of the 18 items, 8 specifically targeted households with children, whereas households without children responded to 10 items. Food security scores were calculated by summing responses to each questionnaire item. Based on these scores, food security status was classified into four groups: food secure (score 0–2), food insecure without hunger (score 3–7 for households with children, score 3–5 for households without children), moderately food insecure with hunger (score 8–12 for households with children, score 6–8 for households without children), and severely food insecure with hunger (score 13–18 for households with children, score 9–10 for households without children). Food-insecure group without hunger refers to a reduced diet quality or variety without decreased food intake. Moderate food-insecure group with hunger indicated uncertainty about acquiring food due to financial constraints, potentially resulting in skipped meals or occasional food shortages. Severe food insecurity with hunger indicated situations where individuals experienced complete food deprivation due to financial limitations, potentially leading to whole days without eating. Food insecurity without hunger, moderate food insecurity with hunger, and severe food insecurity with hunger were collectively classified as the food-insecure group.

### 2.4. Definitions of Metabolically Healthy Obesity and Metabolically Unhealthy Obesity

The definition of MHO varies among studies. In this study, we followed the definition of MHO as stated in the 2022 Korean Obesity Fact Sheet by the Korean Society for the Study of Obesity [24]. The following criteria were used for a diagnosis of MHO: obesity (BMI ≥ 25 kg/m^2^); serum triglycerides ≤150 mg/dL; HDL cholesterol serum concentrations >40 mg/dL (in men) or >50 mg/dL (in women); SBP ≤ 130 mmHg; DBP ≤ 85 mmHg; fasting blood glucose ≤ 100 mg/dL; no medical treatment for diabetes, or hypertension.

Accordingly, MUO was defined when participants with obesity (BMI ≥ 25 kg/m^2^) had any of the following: (1) triglycerides ≥ 150 mg/dL, (2) HDL-cholesterol < 40 mg/dL (men), <50 mg/dL (women), (3) SBP ≥ 135 mmHg, DBP ≥ 85 mmHg, or on treatment for hypertension, (4) fasting glucose ≥ 100 mg/dL or on treatment for diabetes.

### 2.5. Statistical Analysis

Analyses were executed after adjusting for the complex sample design and sample weights. We used a chi-squared test to compare the sociodemographic and lifestyle characteristics of participants with MHO and MUO. Data are presented as unweighted numbers and weighted percentages for each category. Descriptive statistics and Student’s t-tests were used to compare the anthropometric and laboratory profiles of participants with MHO and MUO. The odds ratios (ORs) and 95% confidence intervals (CIs) for factors associated with MHO were calculated using multivariate logistic regression analysis. Furthermore, we calculated the ORs and 95% CIs for food security. Analyses were performed using IBM SPSS Statistics for Windows (version 23.0; IBM Corp., Armonk, NY, USA), and statistical significance was established with two-tailed *p*-values of <0.05.

## 3. Results

### 3.1. Basic Characteristics of the Study Participants with Obesity According to Their Metabolic Health Status

Table 1 presents the participants’ basic characteristics. Of the 5191 participants with obesity, 85.4% (n = 4567) had MUO, and 14.6% (n = 624) had MHO. The proportion of participants with MUO was higher in the older age and higher BMI groups, whereas there was no significant difference based on sex. The proportion of participants with MUO was higher among individuals in the low household income and low education groups than among those in the high household income and high education groups. When considering occupational status, the proportion of participants with MUO was the highest among non-manual workers, followed by those with non-occupation and then manual workers. The proportion of participants with MUO was highest among non-drinkers, followed by heavy drinkers and moderate drinkers. A higher proportion of former smokers had MUO compared to never-smokers or current smokers. The proportion of participants with MUO increased as physical activity decreased.

In terms of dietary behaviors, the prevalence of MUO was higher among individuals who ate out less than once a day than among those who ate out one or more times per day. Similarly, MUO was more prevalent in the low-fat intake group than in the high-fat intake group. Participants with MUO were more likely to be in the food-insecure group. The proportion of MUO increased as food security decreased.

Table 2 lists anthropometric and laboratory profiles of participants with MHO and MUO. Participants with MUO were older and had higher BMI, waist circumference, SBP and DBP, fasting glucose, total cholesterol, and AST and ALT levels than those with MHO. Dietary patterns differed between men and women. Among men, those with MUO had lower protein intake than those with MHO. Women with MUO had lower total calorie intake than those with MHO. Both men and women with MUO consumed higher amounts of carbohydrates and lower amounts of fat than those with MHO.

### 3.2. Factors Associated with Metabolically Unhealthy Obesity

Table 3 presents the ORs and 95% CIs for MUO according to sociodemographic and lifestyle characteristics. After adjusting for age, sex, BMI, household income, education, occupation, alcohol consumption, smoking status, physical activity, eating out, excessive calorie intake, high fat intake, and food security, the OR (95%CI) for MUO increased in participants aged 40–59 years (OR: 2.92, 95% CI: 2.32–3.67) and in those aged ≥60 years (7.15, 5.00–10.23) compared to those with age 19–39 years. The odds for MUO were higher among those with BMI ≥30.0 kg/m^2^ (3.80, 2.60–5.57) and <12 years of education (1.58, 1.08–2.31) compared to those with BMI <30.0 kg/m^2^ and ≥12 years of education, respectively. The odds for MUO significantly increased as physical activity decreased (1.45, 1.03–2.05 for those with moderate activity; 1.95, 1.43–2.66 for those with low activity) compared with those with high physical activity. The odds for MUO were 1.35 (1.03–1.78) among non-manual workers and 1.27 (0.99–1.64) among manual workers compared to those without occupation. Regarding dietary behaviors, the odds for MUO decreased in participants with high fat intake (0.64, 0.50–0.82). Supplementary Table S1 shows the ORs and 95% CIs for MUO according to sociodemographic and lifestyle characteristics in each sex.

### 3.3. Association Between Food Insecurity and Metabolically Unhealthy Obesity in Adults with Obesity

Table 4 shows OR (95% CIs) for MUO according to food security status. In the crude analysis, the OR (95% CIs) for MUO was 1.98 (1.20–3.28) in the food-insecure group compared to the food-secure group. After adjustment for age, sex, and lifestyle factors, the odds for MUO were 1.87 (1.03–3.43) in the food-insecure group. With extra adjustment of education and occupation, the odds for MUO were 1.85 (1.01–3.28) in the food-secure group. When adjusted for age, sex, lifestyle factors, education, occupation, and household income, the odds for MUO were 1.78 (0.96–3.28). Supplementary Table S2 presents ORs (95% CIs) for MUO according to food security status in each sex.

## 4. Discussion

This study showed that older age, higher BMI, lower educational level, lower fat intake, non-manual workers, and lower physical activity were associated with MUO. Additionally, food insecurity was associated with MUO.

As the prevalence of MUO among individuals with obesity varies depending on the definitions and criteria used in different studies, direct comparison of the percentages of MUO across studies may not be possible. The percentage of US adults with MHO among individuals with obesity was 15% during 2015–2018 [25]. A meta-analysis of 19 studies found that approximately 35% of individuals with obesity were metabolically healthy, although this percentage varied across countries [26]. Studies by Liu et al. and Zoghi et al. show that differences in the definition of MUO lead to variations in its prevalence [9,27]. For example, one study reported that the prevalence of MHO:MUO can range from 4.2%:20.1% to 13.6%:10.6%, depending on the specific criteria used. Studies from South Korea revealed that the prevalence of MHO ranged from 5.7% to 25.8%, while that of MUO varied between 13.6% and 25.9% [9].

The association of MUO with age and BMI in this study aligns with the findings of previous research on metabolic states. Aging changes the composition and function of adipose tissue, leading to insulin resistance and abnormal lipid storage [28]. As people age, there is a reduction in brown adipose tissue activity, a decline in sex hormone levels, and an increase in abdominal fat due to lipid redistribution from subcutaneous to visceral areas [28]. These alterations in adipose tissue affect the secretion of hormones called adipokines, thereby promoting chronic, low-grade systemic inflammation [28]. This finding is consistent with the study of Zoghi et al. [9]. Obesity accelerates the aging process by increasing inflammation and the risk of age-related diseases, and vice versa. A higher BMI leads to various obesity-related comorbidities. For instance, a study by Prospective Studies Collaboration showed that every 5-unit increase in BMI was associated with a 40% higher risk of ischemic heart disease mortality, along with the findings that BMI is strongly associated with diabetes [29]. In previous studies, being male was associated with MUO [9,10]; however, there was no association between gender and MUO in this study. Unlike the finding in this study, one study of Korean young adults showed that obese young men were more likely to have obesity-related comorbidities such as abdominal obesity, hypertension, hypercholesterolemia, and hypertriglyceridemia than obese young women [30].

Lower educational attainment was associated with a higher risk of MUO in this study, while previous studies showed an association between low socioeconomic status and risk of MUO. This finding is consistent with Annie’s study, which showed that poor health is linked to factors such as low income, limited resources, unhealthy behaviors, living in unhealthy neighborhoods, and other socioeconomic factors; poor health, in turn, contributes to educational setbacks and hinders schooling by causing learning disabilities, absenteeism, and cognitive disorders [31]. Education, on the other hand, helps promote and sustain healthy lifestyles and positive choices, fosters positive relationships, and improves well being at the personal, family, and community levels. It also encourages greater attention to preventive care [32]. Among the various levels of adult education, tertiary education is the most crucial indicator because it significantly influences health outcomes, such as reducing infant mortality, increasing life expectancy, improving child vaccination rates, and boosting school enrollment rates [32]. Therefore, education could be a way to supplement health policies for better health literacy, healthier lifestyle choices, and increased awareness of nutrition and physical activity.

In the present study, lower physical activity was associated with MUO, but smoking and heavy drinking were not associated with MUO. The relationship between physical activity and body fat levels is well documented. Physical activity plays a critical role in energy balance and metabolism by directly affecting body composition, including fat mass [33]. It also influences hormone levels in the body, such as insulin and cortisol, which are important for the regulation of fat storage and mobilization [34]. Exercise improves insulin sensitivity, reducing the risk of fat accumulation, particularly around the abdomen [35]. Engaging in regular physical activity can also affect appetite and satiety hormones, potentially leading to better regulation of food intake and prevention of overeating, which is crucial for maintaining a healthy body fat percentage [36]. Exercise can also improve mood and reduce stress, which can indirectly affect body fat levels by reducing emotional eating or binge eating tendencies [37]. In previous studies, lifestyle factors such as smoking and heavy drinking were also associated with MUO, in addition to low physical activity [9,10].

This finding also accounts for the association between non-manual work and MUO, demonstrating the impact of sedentary work environments on health. The association between sedentary behavior and metabolic syndromes is quite certain [38]. A meta-analysis by Ortega et al. showed that individuals with MHO are more active, spend less time on sedentary behaviors, and have better cardiorespiratory fitness than individuals with MUO [39]. As lowering sitting time and increasing physical activity are essential for metabolic health, engaging in physical activity during spare time should be emphasized [40,41]. Policies that encourage physical activity—for instance, subsidizing gymnasium memberships, creating more public parks and recreational facilities, and incentivizing companies to offer wellness programs that promote regular movement and exercise breaks during the workday—should be implemented.

Contrary to previous studies, lower fat intake was linked to MUO in this study, potentially because lower fat intake may, conversely, increase the intake of unhealthy carbohydrates. A low-fat diet may lead to a high-carbohydrate diet, which adversely affects weight, HDL cholesterol, and triglyceride profiles [42]. The Korean diet is known to be a carbohydrate-rich diet, emphasizing the authenticity of this hypothesis. On one hand, ‘bread, potatoes, and sweet snacks’ were associated with MUO, whereas ‘fruit, vegetables, and fish’ were associated with MHO in a Dutch study [43]. Several factors, including country and ethnicity, may influence dietary patterns associated with MUO. Nevertheless, a well-proportioned intake of macronutrients should be encouraged to promote metabolic health in individuals with obesity.

The association between food insecurity and MUO was significant when adjusted for lifestyles; however, it became insignificant after additional adjustments for household income were made in this study. Previous studies have consistently shown that food insecurity is linked to poor metabolic health outcomes, including a higher prevalence of obesity. Research suggests that the stress associated with food insecurity, combined with limited access to nutritious food, leads individuals to consume lower-quality diets that are high in calories but low in essential nutrients, contributing to obesity and related metabolic disorders [44]. Food insecurity does not necessarily mean hunger; rather, it is associated with poor diets, not lower energy intake [45]. Unhealthy food environments may lead to poor dietary choices; energy-dense, nutrient-poor foods are cheaper and more accessible, and low-income neighborhoods are often located in food deserts or food swamps [46]. Specifically, dietary choices in populations with food insecurity include low consumption of fruits and vegetables, lean proteins, and milk products, high consumption of energy-dense, nutrient-poor foods, and low dietary diversity [45,46,47]. Food insecurity may also lead to weight gain through overeating when access to food is unlimited [48].

In addition to food assistance, diverse approaches to improve food security are important. Creating a healthy food environment by improving access to food that is available and affordable contributes to a healthy diet. In Canada, sugar tax and subsidies for fruit and vegetables showed positive health and economic outcomes for low-income groups [49]. The Supplemental Nutrition Assistance Program (SNAP), which provides monthly benefits on an Electronic Benefit Transfer card used to buy food, is the representative federal food assistance program and has also been shown to reduce food insecurity effectively [50]. Bringing in supermarkets and fresh food outlets could develop a healthy food environment [51,52]. As anxiety and stress associated with food insecurity are major contributors to obesity, a safety net such as unemployment insurance to provide stable access to food should also be considered [53,54]. The overcorrection in Model 4 implies a close relationship between food insecurity and low household income. Food insecurity is strongly correlated with low income, as individuals and families with limited financial resources are more likely to struggle with consistent access to adequate and healthy food [55]. To effectively address the issue of food insecurity and its impact on metabolic health, not only improving food security but also addressing the underlying economic conditions that contribute to food insecurity among low-income populations is crucial.

As this study was based on data from KNHANES, it has several inherent limitations. First, as the KNHANES is primarily a cross-sectional survey, data are collected at a single time point, which restricts the ability to establish causality or determine the temporal sequence of events. Therefore, caution is advised when interpreting the results as a causal relationship between MHO and other factors cannot be confirmed within the framework of this study. Additionally, the survey relies on self-reported data for certain variables, introducing potential recall and social desirability biases that may have affected the accuracy of the findings. Furthermore, the definition of MHO may differ from that used in other countries, making direct comparisons challenging. In this study, we used BMI to define obesity; however, BMI has been widely recognized as an imperfect measure of obesity and a poor direct indicator of body fat. Using other valid indicators of obesity, such as waist-to-hip ratio and body composition metrics, would be a way to overcome this limitation; however, we were not able to perform further analysis because the KNHANES do not contain such data.

## 5. Conclusions

Our study showed associations between MUO and age, BMI, educational level, fat intake, occupation, physical activity, and food insecurity. As MHO might be a transient state of MUO, along with the development of patients’ guidelines, public health strategies focusing on improving food security and encouraging physical activity, better education, and appropriate dietary fat intake should be implemented.

## Figures and Tables

**Table 1:** Comparison of sociodemographic and lifestyle characteristics between participants with metabolically healthy obesity and metabolically unhealthy obesity.

	MHO	MUO	*p*-Value
	N (%)	N (%)	
Age group (years)			
19–39	314 (28.1)	848 (71.9)	<0.001
40–59	229 (11.3)	1641 (88.7)	
≥60	81 (4.1)	2078 (95.9)	
Sex			
Men	329 (15.1)	2309 (84.9)	0.348
Women	295 (13.9)	2258 (86.1)	
BMI (kg/m^2^)			
25.0–29.9	576 (16.3)	3739 (83.7)	<0.001
≥30.0	48 (7.0)	815 (93.0)	
–	–	–	–
1st quartile	66 (8.9)	999 (91.1)	<0.001
2nd quartile	154 (13.1)	1169 (86.9)	
3rd quartile	187 (16.2)	1204 (83.8)	
4th quartile	213 (17.0)	1180 (83.0)	
Education (years)			
<12	64 (4.8)	1487 (95.2)	<0.001
≥12	533 (17.7)	2778 (82.3)	
Occupation			
Non-occupation	211 (15.9)	1636 (84.1)	0.006
Manual worker	203 (12.4)	1659 (87.6)	
Non-manual worker	180 (16.9)	966 (83.1)	
Alcohol consumption			
Non-drinker	127 (9.9)	1460 (90.1)	<0.001
Moderate drinker	396 (18.1)	2229 (81.9)	
Heavy drinker	98 (11.3)	827 (88.7)	
Smoking status			
Never-smoker	364 (16.0)	2516 (84.0)	0.041
Former smoker	132 (12.3)	1213 (87.7)	
Current smoker	125 (15.0)	782 (85.0)	
Physical activity			
Low	295 (11.6)	2845 (88.4)	<0.001
Moderate	205 (17.5)	1150 (82.5)	
High	96 (27.7)	275 (72.3)	
Eating out			
<1 time/day	440 (13.6)	3635 (86.4)	0.006
≥1 time/day	184 (17.6)	932 (82.4)	
Excessive calorie intake			
No	544 (14.7)	4024 (85.3)	0.674
Yes	80 (14.0)	540 (86.0)	
High-fat intake			
No	448 (12.4)	3945 (87.6)	<0.001
Yes	176 (25.0)	619 (75.0)	
Food security			
Food-secure group	604 (14.9)	4303 (85.1)	0.046
Food-insecure group without hunger	17 (8.5)	211 (91.5)	
Moderate food-insecure group with hunger	3 (7.7)	44 (92.3)	
Severe food-insecure group with hunger	0 (0.0)	9 (100.0)	

MHO, metabolically healthy obesity; MUO, metabolically unhealthy obesity; BMI, body mass index.

**Table 2:** Anthropometric and laboratory profiles of participants with metabolically healthy obesity and metabolically unhealthy obesity.

	Men			Women		
	MHO	MUO	*p*-Value	MHO	MUO	*p*-Value
	Mean (SE)	Mean (SE)		Mean (SE)	Mean (SE)	
Age (years)	34.7 (0.7)	48.3 (0.4)	<0.001	43.0 (0.9)	54.9 (0.5)	<0.001
Height (cm)	175.0 (0.4)	172.1 (0.2)	<0.001	159.4 (0.4)	157.0 (0.2)	<0.001
Weight (kg)	83.9 (0.6)	83.0 (0.3)	0.172	68.5 (0.4)	69.8 (0.3)	0.014
BMI (kg/m^2^)	27.4 (0.1)	28.0 (0.7)	<0.001	26.9 (0.1)	28.3 (0.1)	<0.001
WC (cm)	92.1 (0.4)	96.2 (0.2)	<0.001	86.9 (0.4)	92.2 (0.2)	<0.001
SBP (mmHg)	115.0 (0.5)	125.1 (0.3)	<0.001	110.1 (0.6)	124.5 (0.4)	<0.001
DBP (mmHg)	73.6 (0.4)	81.4 (0.3)	<0.001	71.3 (0.4)	76.5 (0.3)	<0.001
Fasting glucose (mg/dL)	90.7 (0.3)	110.0 (0.7)	<0.001	91.0 (0.3)	108.1 (0.7)	<0.001
Total cholesterol (mg/dL)	189.6 (1.9)	195.2 (0.9)	0.007	199.0 (2.2)	192.2 (1.0)	0.005
AST (IU/L)	25.4 (0.8)	29.3 (0.4)	<0.001	21.6 (0.6)	25.7 (0.4)	<0.001
ALT (IU/L)	30.5 (1.6)	38.0 (0.7)	<0.001	18.7 (0.7)	25.5 (0.6)	<0.001
Daily dietary intake						
Total calorie (kcal)	2331.7 (62.2)	2250.7 (23.9)	0.216	1688.5 (41.0)	1519.5 (17.0)	<0.001
Carbohydrate (%)	52.7 (0.9)	56.9 (0.4)	<0.001	58.5 (0.8)	63.3 (0.3)	<0.001
Fat (%)	26.4 (0.6)	21.7 (0.2)	<0.001	24.7 (0.6)	20.5 (0.2)	<0.001
Protein (%)	16.3 (0.4)	15.1 (0.1)	0.002	15.0 (0.3)	14.8 (0.1)	0.662

MHO: metabolically healthy obesity, MUO: metabolically unhealthy obesity, SE: standard error, BMI: body mass index, WC: waist circumference, SBP: systolic blood pressure, DBP: diastolic blood pressure, AST: aspartate aminotransferase, ALT: alanine aminotransferase.

**Table 3:** Odds ratios and 95% confidence intervals for metabolically unhealthy obesity according to sociodemographic and lifestyle characteristics.

	Crude	Multi-Adjusted
	OR (95% CI)	* OR (95% CI)
Age group (years)		
19–39	1.00	1.00
40–59	3.06 (2.48–3.78)	2.92 (2.32–3.67)
≥60	9.13 (6.81–12.23)	7.15 (5.00–10.23)
Sex		
Women	1.00	1.00
Men	0.91 (0.75–1.11)	1.26 (0.96–1.65)
BMI (kg/m^2^)		
25.0–29.9	1.00	1.00
≥30.0	2.59 (1.82–3.68)	3.80 (2.60–5.57)
Household income		
4th quartile	1.00	1.00
3rd quartile	1.06 (0.82–1.37)	0.95 (0.72–1.26)
2nd quartile	1.36 (1.06–1.75)	0.99 (0.75–1.30)
1st quartile	2.09 (1.47–2.96)	1.14 (0.77–1.69)
Education (years)		
≥12	1.00	1.00
<12	4.30 (3.18–5.82)	1.58 (1.08–2.31)
Occupation		
Non-occupation	1.00	1.00
Non-manual worker	0.93 (0.74–1.17)	1.35 (1.03–1.78)
Manual worker	1.34 (1.05–1.71)	1.27 (0.99–1.64)
Alcohol consumption		
Non-drinker	1.00	1.00
Moderate drinker	0.50 (0.39–0.64)	0.80 (0.59–1.08)
Heavy drinker	0.86 (0.63–1.20)	1.17 (0.78–1.74)
Smoking status		
Never-smoker	1.00	1.00
Former smoker	1.26 (0.93–1.71)	1.16 (0.85–1.58)
Current smoker	0.93 (0.71–1.20)	1.25 (0.90–1.75)
Physical activity		
High	1.00	1.00
Moderate	1.81 (1.31–2.50)	1.45 (1.03–2.05)
Low	2.91 (2.17–3.90)	1.95 (1.43–2.66)
Eating out		
<1 time/day	1.00	1.00
≥1 time/day	0.73 (0.59–0.91)	0.96 (0.75–1.24)
Excessive calorie intake		
No	1.00	1.00
Yes	1.06 (0.80–1.42)	1.11 (0.80–1.54)
High-fat intake		
No	1.00	1.00
Yes	0.43 (0.34–0.53)	0.64 (0.50–0.82)

OR: odds ratio, CI: confidence interval, BMI: body mass index. * Adjusted for age group, sex, BMI, household income, education, occupation, alcohol consumption, smoking status, physical activity, eating out, excessive calorie intake, and high-fat intake

**Table 4:** Odds ratios and 95% confidence intervals for metabolically unhealthy obesity according to food security status.

	OR (95% CI)
Model 1	
Food-secure group	1.00
Food-insecure group	1.98 (1.20–3.28)
Model 2	
Food-secure group	1.00
Food-insecure group	1.87 (1.03–3.43)
Model 3	
Food-secure group	1.00
Food-insecure group	1.85 (1.01–3.38)
Model 4	
Food-secure group	1.00
Food-insecure group	1.78 (0.96–3.28)

OR, odds ratio; CI, confidence interval. Model 1: crude. Model 2: Adjusted for age, sex, and lifestyle factors (alcohol consumption, smoking status, physical activity, frequency of eating out, excessive calorie intake, and high fat intake). Model 3: Adjusted for age, sex, lifestyle factors (alcohol consumption, smoking status, physical activity, frequency of eating out, excessive calorie intake, and high fat intake), education, and occupation. Model 4: Adjusted for age, sex, lifestyle factors (alcohol consumption, smoking status, physical activity, frequency of eating out, excessive calorie intake, and high fat intake), education, occupation, and household income.

## Data Availability

The data presented in this study are openly available on the website of the Korea Centers for Disease Control and Prevention at https://knhanes.kdca.go.kr/knhanes/sub03/sub03_02_05.do (accessed on 15 January 2024).

## Supplementary Materials

The following supporting information can be downloaded at: https://www.mdpi.com/article/10.3390/nu16223833/s1, Supplementary Table S1. Odds ratios and 95% confidence intervals for metabolically unhealthy obesity according to so-ciodemographic and lifestyle characteristics in men and women with obesity. Supplementary Table S2. Odds ratios and 95% confidence intervals for metabolically unhealthy obesity according to food security status in men and women with obesity
