# Supplementary material for: Factors Associated with Metabolically Unhealthy Obesity and Its Relation to Food Insecurity in Korean Adults with Obesity
Source: Nutrients. 2024 Nov 8;16(22):3833. doi: 10.3390/nu16223833 (PMC11597602; doi:10.3390/nu16223833)
Supplement: Supplementary file 1 [file nutrients-16-03833-s001.zip › nutrients-3272041-supplementary.pdf]

Supplementary Table S1. Odds ratios and 95% confidence intervals for metabolically unhealthy obesity according to so-ciodemographic and lifestyle characteristics in men and women with obesity

	Men		Women	
	Crude	*Multi—adjusted	Crude	*Multi—adjusted
	OR (95% CI)	OR (95% CI)	OR (95% CI)	OR (95% CI)
Age group (years)				
19 – 39	1.00	1.00	1.00	1.00
40 – 59	4.06(3.03—5.44)	3.31(2.40—4.57)	2.02(1.43—2.85)	2.13(1.42—3.19)
≥ 60	12.63(8.22—19.4)	10.254(6.13—17.14)	7.06(4.80—10.38)	4.37(2.57—7.44)
BMI (kg/m^2)				
25.0 – 29.9	1.00	1.00	1.00	1.00
≥ 30.0	2.00(1.26—3.12)	2.81(1.66—4.75)	3.95(2.33—6.70)	6.58(3.85—11.25)
Household income				
4th quartile	1.00	1.00	1.00	1.00
3rd quartile	1.15(0.84—1.60)	1.01(0.77—1.57)	0.89(0.61—1.32)	0.72(0.47—1.01)
2nd quartile	1.56(1.11—2.18)	1.30(0.88—1.90)	1.11(0.77—1.60)	0.66(0.44—1.01)
1st quartile	1.64(1.04—2.58)	1.26(0.77—2.07)	2.62(1.58—4.35)	1.10(0.60—2.02)
Education (years)				
≥ 12	1.00	1.00	1.00	1.00
< 12	5.66(3.14—10.20)	1.43(0.72—2.85)	4.06(2.83—5.83)	1.83(1.13—2.96)
Occupation				
No occupation	1.00	1.00	1.00	1.00
Non—manual worker	1.34(0.97—1.86)	1.87(1.26—2.80)	1.02(0.74—1.41)	0.89(0.60—1.31)
Manual worker	1.79(1.2—2.50)	1.71(1.19—2.45)	0.577(0.41—0.81)	0.96(0.67—1.36)
Alcohol consumption				
Non—drinker	1.00	1.00	1.00	1.00
Moderate drinker	0.54(0.37—0.80)	0.84(0.52—1.35)	0.50(0.36—0.67)	0.78(0.54—1.13)
Heavy drinker	1.25(0.79—1.97)	1.45(0.84—2.52)	0.44(0.28—0.67)	0.80(0.49—1.32)
Smoking status				
Never smoker	1.00	1.00	1.00	1.00
Former smoker	1.30(0.95—1.79)	0.90(0.63—1.27)	1.02(0.50—2.02)	0.82(0.38—1.78)
Current smoker	0.63(0.46—0.87)	0.78(0.53—1.14)	1.44(0.818—2.53)	1.01(0.52—1.95)
Physical activity				
High	1.00	1.00	1.00	1.00
Moderate	1.81(1.21—2.69)	1.53(1.00—2.35)	1.95(1.11—3.41)	1.32(0.73—2.38)
Low	2.72(1.92—3.85)	1.82(1.26—2.65)	3.47(2.03—5.94)	2.02(1.14—3.58)
Eating out				
< 1 time/day	1.00	1.00	1.00	1.00
≥ 1 time/day	0.76(0.59—0.98)	0.89(0.66—1.18)	0.67(0.45—0.99)	1.10(0.69—1.76)
Excessive calorie intake				
No	1.00	1.00	1.00	1.00
Yes	1.09(0.80—1.50)	1.02(0.72—1.47)	1.11(0.59—2.07)	1.16(0.56—2.40)
High fat intake				
No	1.00	1.00	1.00	1.00
Yes	2.43(1.82—3.24)	1.56(1.13—2.148)	2.22(1.59—3.08)	1.44(0.99—2.10)

OR: odds ratio, CI: confidence interval, BMI: body mass index. *Adjusted for age group, sex, BMI, household income, education, occupation, alcohol consumption, smoking status, physical activity, eating out, excessive calorie intake, and high-fat intake

Supplementary Table S2. Odds ratios and 95% confidence intervals for metabolically unhealthy obesity according to food security status in men and women with obesity

	Men	Women
	OR (95% CI)	OR (95% CI)
Model 1		
Food secure group	1.00	1.00
Food insecure group	2.28(1.02—5.11)	1.73(0.89—3.35)
Model 2		
Food secure group	1.00	1.00
Food insecure group	1.78(0.75—4.25)	2.12(0.97—4.64)
Model 3		
Food secure group	1.00	1.00
Food insecure group	1.90(0.80—4.50)	1.97(0.90—4.34)
Model 4		
Food secure group	1.00	1.00
Food insecure group	1.80(0.75—4.32)	1.83(0.82—4.10)

OR, odds ratio; CI, confidence interval. Model 1: crude. Model 2: Adjusted for age, sex, and lifestyle factors (alcohol consumption, smoking status, physical activity, frequency of eating out, excessive calorie intake, and high fat intake). Model 3: Adjusted for age, sex, lifestyle factors (alcohol con-sumption, smoking status, physical activity, frequency of eating out, excessive calorie intake, and high fat intake), education, and occupation. Model 4: Adjusted for age, sex, lifestyle factors (alcohol consumption, smoking status, physical activity, frequency of eating out, excessive calorie intake, and high fat intake), education, occupation, and household income.
